# Mapping glucose-induced hemodynamics in white fat depots with label-free optoacoustics

**DOI:** 10.1016/j.pacs.2025.100793

**Published:** 2025-12-23

**Authors:** Nikolina-Alexia Fasoula, Nikoletta Katsouli, Michael Kallmayer, Vasilis Ntziachristos, Angelos Karlas

**Affiliations:** a Chair of Biological Imaging, Central Institute for Translational Cancer Research (TranslaTUM), School of Medicine and Health, Technical University of Munich, Munich, Germany; b Institute of Biological and Medical Imaging, Bioengineering Center, Helmholtz Zentrum München, Neuherberg, Germany; c Technical University of Munich, School of Medicine and Health, Clinic and Polyclinic for Vascular and Endovascular Surgery, TUM University Hospital, Munich, Germany; d Clinic for Vascular Surgery, Helios Klinikum München West, Munich, Germany; e DZHK (German Centre for Cardiovascular Research), partner Site Munich Heart Alliance, Munich, Germany; f Chair for Computer Aided Medical Procedures & Augmented Reality, Technical University of Munich, Munich, Germany

**Keywords:** Photoacoustics, Cardiovascular disease, Obesity, Hyperglycemia, Postprandially, White adipose tissue

## Abstract

Subcutaneous adipose tissue (SAT) hemodynamics is an indicator of cardiometabolic health. Herein, we demonstrate a non-invasive approach for imaging SAT hemodynamics in humans using multispectral optoacoustic tomography (MSOT). We evaluated different SAT depots in individuals with low (< 24 kg/m²) and high (≥ 24 kg/m²) BMI, with each group consisting of 8 participants, during oral glucose challenges. Our results indicate a significant decrease in glucose-induced hyperemic responses within SAT for individuals with higher BMI, at 60 min postprandially. MSOT also revealed that abdominal SAT exhibited a more active hemodynamic status compared to femoral SAT in both groups when compared to baseline measurements. MSOT readouts were further validated against longitudinal blood tests of triglycerides, glucose, lactate, and cholesterol. We introduce MSOT as a new method for studying SAT hemodynamics across multiple depots in a single test, providing invaluable insights into SAT physiology related to BMI fluctuations and general cardiometabolic health.

## 1 Introduction

The pandemic of cardiometabolic diseases, such as obesity, diabetes, and cardiovascular disease, has prompted increased exploration of the role of white adipose tissue (WAT) in these conditions [1]. WAT is typically divided into visceral adipose tissue (VAT) and subcutaneous adipose tissue (SAT), with the latter making up 85 % of the total body WAT [2]. SAT changes, whether structural or functional, have already been linked to cardiometabolic diseases. For instance, the accumulation of SAT in the abdominal area has been strongly associated with a high risk of developing diabetes and cardiovascular disease [3], [4]. Besides structural changes, functional SAT changes have also been observed. Specifically, SAT hemodynamic responses to stimuli, such as oral glucose loading, have been reported to be impaired in individuals with obesity and diabetes [5]. These findings make SAT and its hemodynamics an excellent target for investigating disease pathophysiology or patient monitoring. As a result, various techniques have been developed to assess SAT hemodynamics. The most common technique involves tracking the washout rate of ^133^Xe using a scintillation counter system [6], [7]. ^133^Xe is typically injected locally into the abdominal SAT before monitoring [6], [7]. This method is simple, quantitative, and sensitive to small changes. However, it has disadvantages, including the need for a radioactive isotope (albeit at a low dose), the invasive and painful injection required, and the inability to measure different positions of SAT concurrently (e.g., abdominal, gluteal, and femoral) [8], [9]. It would be beneficial to have the option to measure different positions of SAT simultaneously, given the different roles of SAT depots in general metabolism.

Another technique is based on ethanol microdialysis, which is also easy to perform in humans and avoids the use of a radioactive isotope [10]. It combines microdialysis with monitoring the washout of an injected marker (ethanol dilution technique). The calculated "ethanol ratio" depends on blood flow: the greater the adipose tissue blood flow (ATBF), the more ethanol is removed from SAT per unit of time [10]. However, this method is also invasive, as it involves the insertion of a catheter into the measured tissue and requires sufficient skin-fold thickness for successful catheterization [11]. Furthermore, it is less delicate than the ^133^Xe washout method [12]. Both techniques are further limited by low temporal resolution but, most importantly, by the fact that they are only capable of providing one-dimensional information without detailed imaging of the examined tissue region. Moreover, the ATBF measured by the two methods seems to be substantially different, meaning that low accuracy and reliability are existing issues [12].

To overcome the limitations mentioned above, ATBF was also measured using laser-doppler flowmetry (LDF) [13]. Even though LDF is label-free, non-invasive, and has a high temporal resolution, it is also one-dimensional, has limited penetration (less than 1 cm), and provides a low spatial resolution of several mm^3^: a tissue volume that may well include a non-homogeneous mixture of tissues (e.g., skin, SAT, muscle) while measuring.

In terms of imaging, positron emission tomography (PET) with [^18^F]-fluoro-deoxy-glucose ([^18^F]-FDG) and [^15^O]-labeled water, which enables the non-invasive quantification of tissue glucose uptake and perfusion, has been used for imaging hemodynamics and metabolism of different fat deposits simultaneously [14]. However, PET employs ionizing radiation and requires bulky and costly equipment/infrastructure, as well as highly specialized personnel. In addition, PET is characterized by low spatial (1–5 mm) and temporal (2–10 s) resolution [15], [16], [17].

Contrast-enhanced ultrasound (CEUS) has also been used to image postprandial SAT hemodynamics in patients with diabetes [18]. Although the cost of the method is moderate and radioactive substances are not needed, it requires the repeated injection of microbubbles (because they remain in the bloodstream only for 3–5 min) during the 60-minute duration of the test. Furthermore, possible, yet rare, allergic reactions might hinder its use.

Thus, current techniques for monitoring SAT hemodynamics have limitations that hinder their widespread use, highlighting the need for new, ideally imaging, techniques. Such techniques could not only improve our understanding of local and global metabolic physiology and pathology in relevant diseases but could also provide additional biomarkers that can be correlated with diseases and potentially used as predictors of disease progression and therapy efficacy in future patients [19].

In this pilot study, we demonstrate the ability to conduct label-free, non-invasive imaging of SAT hemodynamics in humans using optoacoustic imaging and specifically MSOT. Optoacoustic imaging has already demonstrated successful applications within the cardiometabolic field [20], [21], [22]. By using MSOT to measure SAT hemodynamics during an oral glucose challenge in individuals with different body mass indices (BMIs), we found that subjects with higher BMIs exhibited significantly reduced glucose-induced hyperemic SAT responses compared to those with lower BMIs, as expected. Additionally, we demonstrated for the first time the capability of MSOT to simultaneously image the hemodynamics of multiple clinically relevant SAT depots, such as those in the abdominal and gluteofemoral regions. This enabled us to investigate potential differences in temporal reactions among the examined SAT depots. Finally, we validated MSOT-extracted SAT hemodynamics against relevant blood tests (triglycerides, glucose, lactate, and cholesterol). Validating our results via blood tests further emphasizes the reliability of MSOT in assessing SAT hemodynamics, paving the way for additional studies in the cardiometabolic field and advancing MSOT closer to clinical translation.

## 2 Methods

### 2.1 Study design and experimental protocol

We enrolled sixteen (n = 16) participants in total, categorized into two groups: high (≥24 kg/m², n_1_=8) and low (<24 kg/m², n_2_=8) body mass index (BMI). Considering the exploratory and preliminary nature of the current study, we applied the “resource equation” method for the determination of the sample size [23]. According to this method, the value E = Total number of subjects − Total number of groups, which represents the degree of freedom for analysis of variance (ANOVA), should lie between 10 and 20 to demonstrate a sufficient sample size. In our case, *E* = 16–2 = 14, demonstrating that the selected sample size is sufficient.

**Fig. 1:**
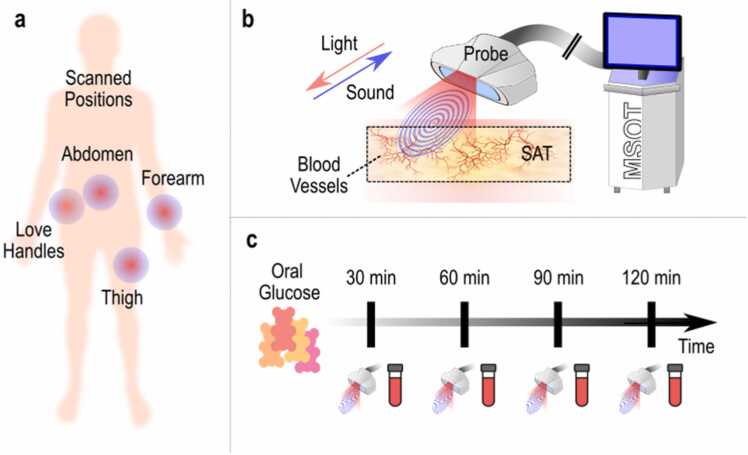
Workflow of the study. (a) Overview of the examined anatomic positions. (b) Principle of operation of MSOT imaging of the SAT over each examined position. (c) Timeline of an examination session. After the consumption of the oral glucose meal, we performed MSOT of the above-mentioned anatomic positions and blood sampling/analysis every 30 min for 2 h in total. SAT: Subcutaneous adipose tissue.

Detailed demographics are provided in Table S1 (see Supplementary Material*)*. All participants were non-smokers and reported no history of cardiometabolic disease. They were kindly instructed to abstain from consuming caffeine, food, and alcohol for a minimum of 12 h prior to the scheduled measurements. Measurements were conducted in a quiet, standardized examination room maintained at a temperature of approximately 23°C. Participants were allowed a rest period of approximately 20 min before undergoing the 2-hour oral glucose challenge. A visual description of the study workflow is provided in Fig. 1.

The ethics committee of the Technical University of Munich approved the study (Protocol #324/21S). The study was conducted in accordance with the World Medical Association Declaration of Helsinki. Before participating in the study, all individuals provided their written informed consent.

### 2.2 Oral glucose test

Following a 12-hour overnight fast, all participants ingested an oral glucose load within a 5-minute window. The composition of the glucose-rich meal was standardized to 50 g of glucose, equivalent to 12 HARIBO©-Gummy Bears (HARIBO™ GmbH & Co. KG, Grafschaft, Germany). In detail, the meal encompassed a total of 50 g of sugar, inclusive of 0.4 g of fat, 0.08 g of saturated fat, 41.5 g of carbohydrates, and 3.6 g of protein, yielding an energy provision of 1186 KJ/279 Kcal. The consumption of the meal aimed at emulating a condensed version of the glucose tolerance test [24]. Notably, the consumption of this glucose load was not restricted to a fasting state and could be undertaken at any point during the day.

#### 2.2.1 Anthropometric measurements

Blood pressure, oxygen saturation levels, and pulse rates were systematically documented in a duplicated manner while in a fasting state, followed by subsequent evaluations every 30 min over the 2 h post-consumption of the oral glucose-rich meal. Blood pressure readings were obtained using an automated sphygmomanometer (M400 Intelli© IT, Omron Healthcare Co., Ltd., Kyoto, Japan) with an appropriate cuff size. These measurements were recorded at baseline in a fasting state and continued at 30-minute intervals throughout the oral glucose challenge.

In addition, a fingerstick blood sample was collected at the onset of the session (fasting state) to assess fasting capillary blood glucose levels, lipid profiles (encompassing total cholesterol and triglycerides), and lactate concentrations utilizing an accredited hand-held point-of-care device (Accutrend® Plus System, Roche Holding AG, Basel, Switzerland). This procedure was repeated every 30 min for the 2-hour interval after the ingestion of the oral glucose meal, totaling five assessments.

We measured participants' body weight using a level balance calibrated to an accuracy of 0.1 kg, with them wearing underwear or light clothing. Height was recorded to the nearest 1 cm. Circumferences of the thigh, waist, and hip were also measured with a precision of 1 cm. The circumference of the waist was delineated at the midpoint between the lower margin of the last detectable rib and the superior border of the iliac crest for all subjects. The measurement of hip circumference was conducted at the point across the buttocks, yielding the maximum circumference for all subjects. Furthermore, the waist-to-hip ratio (WHR), a dimensionless index, was computed by dividing the waist circumference by that of the hip. Thigh circumference was ascertained on the left leg, directly inferior to the gluteal fold. The demographics and clinical characteristics of the included subjects are provided in Table S1.

#### 2.2.2 MSOT system and data acquisition

Measurements were conducted using a hybrid clinical MSOT/US (Acuity©, iThera Medical GmbH, Munich, Germany) used in several previous clinical studies [25], [26], [27], [28]. Ultrasound detection was conducted by 256 piezoelectric elements with a central frequency of 4 MHz arranged in an arc of 145 °. Illumination output, which was mounted on the same hand-held probe, emitted light in short pulses (∼ 10 ns) at a rate of 25 Hz. Each pulse delivered almost 15 mJ of energy over a rectangular area of around 1 cm× 4 cm, a feature that complies with the safety limits of lasers used for medical applications [29]. For multispectral image acquisition, we employed 28 light wavelengths (from 700 to 970 nm at steps of 10 nm). Thus, the recording of a complete set of 28 single-wavelength optoacoustic images lasted ∼1 s. Co-registered US images were recorded in parallel to the MSOT images at a frame rate of ∼8 Hz.

The MSOT hand-held scanning probe was repeatedly placed over the same four positions during the test, guided by stable skin markers. The initial measurement was taken by placing the probe over the abdomen, specifically to the immediate right of the umbilicus; the second over the gluteal region (love handles), the third over the femoral region (thigh) and the fourth one over the forearm (Fig. 1). Recordings of these regions were taken just before the consumption of the oral glucose meal (post-fasting) and then every 30 min and for 2 h after it (5 measurements in total). The 30 min was determined to be a suitable time span to assess postprandial responses. Each position was scanned for ∼20 s.

As previously shown, MSOT images acquired at 750 nm reveal primarily deoxyhemoglobin (Hb) contrast; images at 850 nm reveal contrast primarily from oxyhemoglobin (HbO_2_), whereas images at 800 nm, where the optical absorption of Hb and HbO_2_ are equal (isosbestic point), reveal contrast from total Hb (THb) [30], [31]. MSOT images at the 930 nm show mainly the lipid distribution within the imaged region and serve, thus, as an additional input for anatomic orientation and detection of the SAT region [25], [27], [31], [32].

#### 2.2.3 Data processing and analysis

Acquired MSOT data were reconstructed using a model-based reconstruction method published elsewhere and already used in clinical MSOT applications [33]. One characteristic 800 nm-frame (THb content) was selected per subject for each measurement time point and position. Next, precise regions of interest (ROIs) were manually segmented within the SAT region visualized in the MSOT images. The subcutaneous fat regions were identified in consensus between two clinicians with experience in clinical MSOT and ultrasound imaging. Apart from prior anatomical knowledge, SAT was identified by its characteristic texture in traditional ultrasound (US). SAT identification was further facilitated by its appearance in MSOT, characterized by a high optoacoustic signal compared to other soft tissues at 930 nm because, at this very wavelength, the lipids absorb the most in the NIR [25], [26], [27].

Finally, the mean optoacoustic intensity values of the pixels belonging to the segmented ROIs were used to plot the time course of the THb-signal within the SAT for each anatomic region during the oral glucose challenges (*see Results*). The THb signal calculations took place on the recorded MSOT images without applying any filtering or denoising, a processing step that took place only for visualization purposes in the corresponding figures. In statistical analyses reporting differences or changes, values were reported as median ± median absolute deviation (MAD) [34]. The respective MADs were taken into consideration when calculating the differences between median values. Thus, each relative difference was calculated based on the formula: $\frac{\mathrm{media}n_{1}-\mathrm{media}n_{2}}{\frac{\mathrm{MA}D_{1}+\mathrm{MA}D_{2}}{2}}$. To assess the appropriateness of using the median and median absolute deviation (MAD) for the statistical analysis of our data, we performed Shapiro–Wilk normality tests for the different distributions at each follow-up time point, separately for each BMI group and body position. In this test, W is the Shapiro–Wilk statistic (ranging from 0 to 1), which indicates the degree to which the sample distribution approximates a normal distribution. Values closer to 1 indicate higher normality. The p-value indicates whether deviations from normality are statistically significant (α = 0.05). Because many, though not all, distributions deviated significantly from normality (p < 0.05, see Table S2, Supplementary Material), we used median ± MAD as robust summary statistics.

## 3 Results

**Fig. 2:**
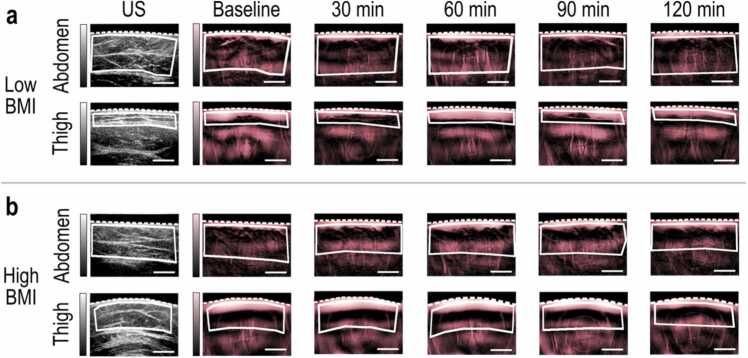
Exemplary MSOT image series of SAT hemodynamics at different time points during the oral glucose. (a) Representative ultrasound (US) and 800 nm-MSOT frames in the abdominal and thigh regions for an individual of low BMI (body mass index). (b) Representative US and 800 nm-MSOT frames in the abdominal and thigh regions for an individual of high BMI. White dashed line: skin, white continuous line: subcutaneous adipose tissue (SAT) region, Scale bars: 1 cm.

As a first step, we investigated the hemodynamic responses of the different SAT depots at several time points after the consumption of the oral glucose meal. Each hemodynamic response is provided as a series of MSOT images captured at 800 nm illumination wavelength, where the THb signal that reports the SAT perfusion is prominent. The MSOT readouts show that, on average, participants exhibit the highest increase (%) of perfusion within the SAT at approximately 60 min after consuming the oral glucose meal (Table S3, see Supplementary Material). In particular, the mean perfusion peak time is 60 min in the abdomen, 63.8 min in the love handles, 56.3 min in the thigh, and 63.8 min in the forearm, yielding a global average of approximately 61.0 min for the lower BMI group. Correspondingly, for the higher BMI group, the mean perfusion peak times are 67.5 min (abdomen), 52.5 min (love handles), 63.8 min (thigh), and 56.3 min (forearm), with a global average of 60.0 min. Thus, the mean observation of approximately 60 min for the mean perfusion peak time within SAT seems to be independent of BMI and the measured SAT depot, indicating a whole-body hemodynamic response of SAT to the glucose meal. The previously described phenomenon is depicted in Fig. 2, which provides an exemplary series of THb-content (perfusion) MSOT images recorded at 800 nm at several time points before (baseline) and after (at 30 min, at 60 min, at 90 min, and 120 min) the consumption of the oral meal for each measured region (abdominal, gluteal – love handles, femoral, forearm) of a participant of each group.

**Fig. 3:**
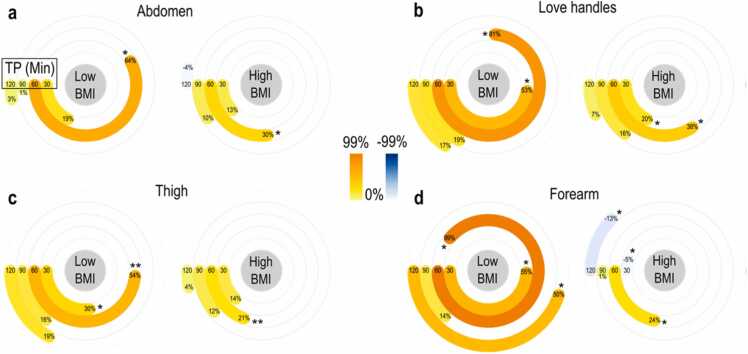
Percentage changes of SAT perfusion measured at different time points over the examined anatomic regions. (a) Abdomen, (b) Love handles, (c) Thigh, and (d) Forearm. Values refer to median percentage changes of the 800 nm-optoacoustic signal measured at 30 min, 60 min, 90 min, and 120 min (TP: time points) after the oral glucose load with reference to the corresponding baseline (see Table S3). Significant differences between the two groups (Low BMI: <24 kg/m² and High BMI: ≥24 kg/m²) were marked with * for p-values < 0.05 or ** for p-values < 0.01.

Next, triggered by previous studies showing that the glucose-induced hemodynamic SAT response might be affected in obesity or metabolic disease, we investigated the possible differences in MSOT-extracted glucose-induced SAT hemodynamics between the two BMI-associated groups [35], [36]. In fact, at 60 min, participants with lower BMI show significantly (p < 0.05) higher perfusion of SAT in all examined anatomic compartments compared to the higher BMI participants (Table S4, see Supplementary Material). In specific, for the lower BMI group, the relative increase (compared to baseline) of the MSOT-extracted THb signal is 64.20 %±36.29 % in the abdomen, 81.24 %±22.93 % in the love handles, 53.86 %±14.59 % in the lower extremity (thigh), and 98.81 %±66.10 % in the upper extremity (forearm). In contrast, for the participants with higher BMI, the corresponding relative changes at 60 min are 30.15 %±10.20 %, 38.12 %±8.83 %, 21.44 %±3.69 %, and 24.10 %±4.43 %, respectively. But apart from the SAT response at 60 min, or else the global mean perfusion peak time as shown in the previous step, the difference between the two groups is proven to be statistically also significant at 30 min after the consumption of the meal within the love handles, thigh, and forearm regions after 30 min (p-value=0.0274, 0.0274, and 0.0415, respectively). The corresponding statistical analysis reported by the median percentage change compared to the baseline for the two groups is provided in Fig. 3 (see also Table S4).

It is known that the SAT of the abdominal region plays a different role not only in postprandial but also in general metabolism compared to the SAT of the gluteofemoral region. In particular, the latter seems to have a protective role against metabolic risk, a property that comes in contrast with the role of the abdominal fat [37]. Such a difference may well be reflected in different perfusion profiles. Thus, as a next step, we explored the possible differences in the MSOT-extracted SAT perfusion between the gluteofemoral and abdominal regions at the baseline, i.e., before the oral consumption of the glucose meal (fasting state). In a per-subject analysis, it appears that in 87.5 % (7 out of 8) of the lower BMI individuals, the abdominal SAT is characterized by higher perfusion/THb values than the femoral area. Correspondingly, only 50 % (4 out of 8) of the higher BMI individuals exhibited this trend. Thus, as a general trend, the expected positive perfusion difference between the two anatomic regions (abdominal vs. femoral) seems to be more frequent in the lower compared to the higher BMI group. Finally, the postprandial relative difference (median difference considering MADs) between the abdominal and femoral regions of THb-content (abdomen-thigh) was at 30 min −1.07 and −0.32 and + 0.41 and + 0.82 at 60 min for the lower and the higher BMI group, correspondingly (see Table S4). The latter indicates a higher increase in the abdominal compared to the femoral SAT postprandially (at least at 60 min, the peak reaction time point), as also shown by previous studies [38].

**Fig. 4:**
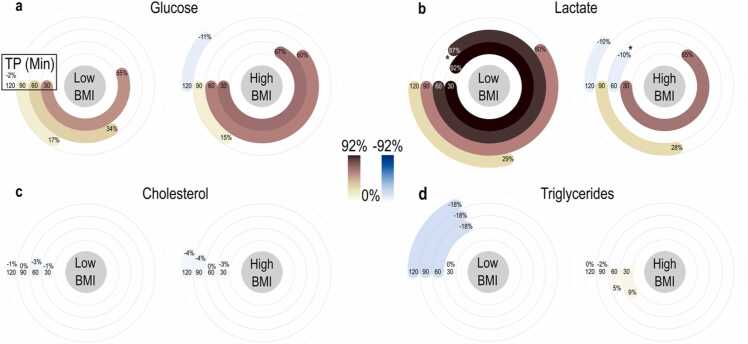
Longitudinal blood analyses during the oral glucose test. Values represent the median percentage changes measured in capillary blood at each time point (TP: 30 min, 60 min, 90 min, and 120 min) after the oral glucose load compared to the baseline. Significant differences between the two groups of low and high BMI are marked with * for p-values < 0.05 (see Table S4). Low BMI: < 24 kg/m² and High BMI: ≥ 24 kg/m².

Blood analyses supported optoacoustic measurements over time during the oral glucose challenge. The recorded parameters were glucose, lactate, cholesterol, and triglycerides. Regarding blood tests, we also focused primarily on the 60-minute and secondarily on the 30-minute time points after the consumption of the oral glucose load, which were the time points with the most prominent differences in the SAT perfusion between the two groups. Individuals with lower BMI exhibit the highest median increase in blood glucose (55.27 %±19.84 %) and lactate (91.89 %±25.00 %) at 30 min after consuming the oral glucose meal. The corresponding increases at 60 min are 33.78 %±23.28 % and 87.30 %±38.71 %, respectively. Conversely, cholesterol levels slightly decreased by (-1.09 %±5.02 %) after 30 min, while triglyceride levels remained unchanged. After 60 min, there is a relative decrease in cholesterol (-3.33 %±3.88 %) and triglycerides (-17.65 %±20.50 %) compared to the baseline. Individuals with higher BMI exhibit higher increases in blood glucose levels (67.18 %±29.91 % at 30 min and 60.35 %±35.54 % at 60 min) compared to the lower BMI group. On the contrary, lactate levels demonstrate a clear yet lower increase (65.00 %±25.33 %) at 30 min and a decrease (-10.42 %±%33.55) at 60 min. Although cholesterol levels slightly decrease (-3.37 %±2.52 %) at 30 min and decrease further by (-0.26 %±2.05 %) at 60 min, triglyceride levels increase by (8.97 %±18.12 %) at 30 min and (5.24 %±11.22 %) at 60 min for this group. As observed, the peak time points for glucose were mainly the 30 min and secondarily the 60 min postprandially. However, despite the trends observed, the differences between the two groups are generally not characterized by statistical significance. In fact, the only statistically significant difference is recorded for the lactate levels at 60 min after the consumption of the oral glucose meal (Fig. 4, Table S5).

## 4 Discussion

SAT hemodynamics are essential for regulating not only local tissue metabolism and nutrient exchange but also general metabolism. On the one side, adequate SAT perfusion is connected to efficient local delivery of nutrients, such as glucose, fatty acids, and oxygen, and the removal of waste products. On the other side, SAT perfusion is critical for global processes, such as energy storage, thermoregulation, and general metabolic homeostasis. Thus, changes in SAT hemodynamics have an impact not only on local metabolic function but also on general metabolic health. Indeed, degradation of SAT hemodynamics is associated with conditions such as insulin resistance and obesity [35], [36]. A diminished postprandial vasodilatory response is especially associated with impaired lipid metabolism and potentially increases susceptibility to cardiovascular disease [39]. Therefore, non-invasive monitoring of SAT hemodynamics could serve as a window into general metabolic monitoring for relevant diseases [40]. So far, SAT hemodynamics are tracked via different invasive or non-invasive methods. The Xenon washout technique involves inhaling Xenon and monitoring how quickly it clears from the tissue to provide quantitative data on circulation. Ethanol-microdialysis makes use of probes to infuse ethanol as a tracer into adipose tissue, allowing the collection of samples to assess local blood flow at a microscale level. CEUS, on the other hand, involves injecting contrast agents to dynamically visualize blood flow patterns in real-time. Laser Doppler flowmetry, utilizing laser light, offers a relative index of perfusion at the tissue surface. These methods, which are characterized by limitations, complement each other to yield a comprehensive understanding of SAT perfusion, which is crucial for understanding its intricate dynamics and implications for metabolic health.

Thus, in this study, we explore the use of MSOT as a novel point-of-care technique to assess SAT hemodynamics in a non-invasive and label-free way. Our findings show that MSOT boasts two key benefits over other non-invasive approaches for measuring in vivo hemodynamics: first, it offers direct molecular information regarding SAT hemodynamics based only on the contrast produced by hemoglobin, and second, it provides an exceptional spatial resolution of less than 300 μm, allowing for detailed tomographic imaging in real-time (25 fps) and different anatomical positions. Apart from the high spatial resolution, MSOT reaches penetration depths of 2–4 cm with an approximate horizontal field of view of 4 cm, enabling the exploration of possible intratissue variations, underscoring its utility in elucidating the complex dynamics of postprandial states. Moreover, although our data acquisition was limited to 30-minute intervals in the present study, the high temporal resolution of MSOT offers the potential for non-invasive exploration of real-time lipid-kinetics phenomena in future studies. Finally, MSOT is a hand-held device that can be easily used for long-lasting examinations, such as the ones following the responses to meal ingestion, without increasing patient or operator inconvenience.

Most importantly, our physiological observations are consistent with established SAT hemodynamic patterns following the oral administration of glucose. Recorded data showed that there was a noticeable increase in signal intensities within the segmented SAT regions after subjects consumed the glucose meal. The increase was recorded around 60 min postprandially. In fact, previous studies have shown that SAT perfusion increases at approximately 30–60 min postprandially after the consumption of an oral glucose load or a mixed meal (carbohydrates, proteins, and fat), and this increase lasts for 2–2.5 h [36], [41]. On the one side, in normoweight subjects, ATBF increases from two- to threefold (and even more) in response to nutritional stimuli in the abdominal, femoral, and forearm muscles at the postprandial state [41], [42], [43]. On the other side, both fasting ATBF and its responsiveness to nutrients are reduced in obese individuals [42]. In our study, differences are shown between the two groups of lower and higher BMI (BMI<24 kg/m² and ≥24 kg/m², respectively). In other words, even without large BMI differences between the two groups, MSOT was capable of detecting the pathophysiological differences in SAT hemodynamics.

Another group of readouts of the current study is provided by the blood analyses following the MSOT measurements. Our analyses showed, as expected, a peak of blood glucose at 30 min in both groups, with the most prominent maintenance of the high blood glucose levels for another 30 min (at 60 min after the oral glucose loading) in the high BMI group. In fact, previous studies showed that such a glucose profile may well be recorded in both normal-weight and obese patients [44], [45]. Such a readout confirms the occurrence of the expected physiological response in the examined participants. Furthermore, the fact that the herein measured blood lipid levels were not characterized by statistically significant differences between the two groups excludes the possibility of having measured a lipid-contaminated’ optoacoustic signal at 800 nm because of the circulating intratissue lipids, even if such absorption would be low at the 800 nm (lipids are characterized by a peak absorption at 930 nm), where the MSOT data were recorded, and light absorption of hemoglobin is potent [25], [26], [27].

Finally, our MSOT readouts showed at 60 min after the consumption of the glucose meal a higher increase in the abdominal than the femoral region for both groups (see Table S4), even if the trend was reversed at 30 min postprandially (the increase in the femoral region was more prominent than in the abdominal one). Past studies showed higher ATBF levels in the abdominal compared to the femoral SAT at the fasting state, along with higher increases postprandially [38]. Thus, the above-mentioned postprandial MSOT readouts, along with the general trend of higher MSOT-extracted THb levels at the baseline, agree with previous observations, further highlighting the potential of MSOT as a novel tool to explore postprandial SAT hemodynamics for vaso-metabolic profiling.

Our study does not come without limitations. Sex is an important biological variable in metabolic and vascular research, because several hormonal factors—particularly estrogen effect—could influence fat distribution, insulin sensitivity, and possibly vascular reactivity [46], [47]. Consequently, some of the outcomes observed in the current study may partially reflect physiological variations related to sex, in addition to BMI alone. However, by examining relatively balanced target groups in terms of sex, such effects are expected to be minimal. Future studies with larger cohorts are necessary to clarify the independent and interactive effects of sex and BMI on the measured outcomes.

Furthermore, the imaging depth achieved by MSOT (2–4 cm based on tissue type) is superior in comparison to other purely optical imaging techniques, albeit it remains inferior to that of conventional clinical modalities such as ultrasonography. Nevertheless, in our study, we were able to access critical positions of the SAT within this depth range, yielding comprehensive hemodynamic data that corroborate existing literature. The integration of advanced light fluence correction methodologies, designed to mitigate the effects of intensity attenuation resulting from scattering and absorption phenomena, promises to enhance the precision of MSOT imaging at greater tissue depths, thereby broadening its spectrum of clinical applicability. For example, rather than providing illumination over a wide area, the system could use a narrow laser beam to scan and capture partial images with each light pulse (single wavelength images). The final image would then be created by summing all the partial images, as shown in [48]. In another study, the authors represent light fluence in the spectral domain and introduce eigenspectra multispectral optoacoustic tomography (eMSOT) [49]. The method accounts for wavelength-dependent attenuation of light, enabling the estimation of blood oxygen saturation (SO2) in phantoms or deep tissues of animal models using preclinical MSOT imaging*.* Such techniques, if properly adapted, could compensate for variations in wavelength-dependent fluence. Of course, the implementation and integration of such complex techniques into the clinical MSOT imaging pipeline should be done with absolute accuracy via targeted studies that lie beyond the scope of the current study. The main challenges include the high heterogeneity of human tissues and, thus, their essentially unknown optical properties in situ due to the large variations among individuals, anatomical sites, and disease states, which makes it difficult to apply a single, generic fluence correction model. For example, when conducting SAT studies, it is essential to consider its unique characteristics. SAT is relatively homogeneous and is located just beneath the skin, which has a low thickness ranging from 0.5 to 4 mm. Most importantly, the blood supply to SAT decreases with depth, as deeper tissues are further away from the perforator vessels in the skin that supply blood to the SAT [50].

While our findings suggest a potential association between impaired SAT hemodynamics and insulin resistance, they do not provide direct confirmation of this association. To further explore this potential association, future targeted studies should include direct assessments of insulin sensitivity, such as the hyperinsulinemic-euglycemic clamp test or the estimation of the Homeostatic Model Assessment for Insulin Resistance (HOMA-IR) index [51], [52].

Finally, the number of individuals included in the current study is limited. The primary objective of this research was to furnish a proof-of-concept through a human pilot study, encompassing a modest cohort of participants categorized into lower and higher BMI groups. Future research endeavors necessitate more expansive studies, incorporating larger cohorts of both healthy volunteers and subjects classified as obese, coupled with concurrent blood analyses and the comparison with other benchmark techniques (e.g., ^133^Xe washout method, regarded as the gold standard) to further refine the utility of MSOT in the non-invasive monitoring of SAT hemodynamics.

Herein, we present MSOT as an innovative tool for the non-invasive evaluation of SAT hemodynamics in vivo following the ingestion of an oral glucose challenge. Our findings confirm the main physiological observations in glucose-induced postprandial SAT hemodynamics in unprecedented detail and point-of-care manner using MSOT, further boosting the clinical translation of MSOT as a quintessential instrument for foundational and clinical investigations within the cardio- and vaso-metabolic domains and herald, thus, new avenues for the diagnostics and risk stratification of cardiovascular and metabolic diseases.

## CRediT authorship contribution statement

**Nikolina-Alexia Fasoula:** Writing - original draft, Visualization, Project administration, Investigation, Formal analysis, Data curation, Conceptualization. **Nikoletta Katsouli:** Writing - original draft, Visualization, Software, Methodology, Formal analysis, Data curation. **Michael Kallmayer:** Writing - review & editing, Resources. **Vasilis Ntziachristos:** Writing - review & editing, Resources, Funding acquisition. **Angelos Karlas:** Writing - original draft, Visualization, Resources, Project administration, Investigation, Funding acquisition, Formal analysis, Data curation, Conceptualization.

## Declaration of Competing Interest

Vasilis Ntziachristos is a founder and equity owner of Maurus OY, sThesis GmbH, Spear UG, Biosense Innovations P.C., and I3 Inc. The other authors declare that they have no known competing financial interests or personal relationships that could have appeared to influence the work reported in this paper.

## Data Availability

Data will be made available on request.
