# Supplementary material for: Mapping glucose-induced hemodynamics in white fat depots with label-free optoacoustics
Source: Photoacoustics. 2025 Dec 23;47:100793. doi: 10.1016/j.pacs.2025.100793 (PMC12859229; doi:10.1016/j.pacs.2025.100793)
Supplement: Supplementary file 1 — Supplementary material [file mmc1.docx]

**Supplementary Material**

**Table S1**. Population demographics and clinical characteristics.

|  | **N** | **Mean Age ± SD (years)** | **Sex (M/F)** | **Mean BMI ± SD (Kg/m^2^)** | **SBP ±**  **SD (mmHg)** | **Mean Waist**  **Circumference ± SD (cm)** | **Mean Hip**  **Circumference ± SD (cm)** | **Mean Thigh**  **Circumference**  **± SD (cm)** | **Mean WHR**  **± SD** |
| --- | --- | --- | --- | --- | --- | --- | --- | --- | --- |
| **Low**  **BMI** | 8 | 28.3 ±  5.1 | 5/3 | 21.5 ±  1.7 | 112.6 ±  10.5 | 77.2 ±  8.6 | 101.4 ±  7.6 | 50.0 ±  3.3 | 0.76 ±  0.06 |
| **High**  **BMI** | 8 | 34.4 ±  11.3 | 3/5 | 26.0 ±  2.0 | 123.7 ±  9.6 | 91.9 ±  8.7 | 109.1 ±  10.5 | 57.8 ±  6.5 | 0.84 ±  0.08 |
| Low BMI: <24 kg/m², High BMI: ≥ 24kg/m² | | | | | | | | | |

**Table S2.** Shapiro–Wilk normality tests for the different distributions at each follow-up time point, for each BMI group and anatomic position.

| BMI | Position | Time | W | p-value | Normality |
| --- | --- | --- | --- | --- | --- |
| ≥24 kg/m² | Abdomen | 30 | 0.5619 | 0.0001 | Non-normal |
| ≥24 kg/m² | Abdomen | 60 | 0.7070 | 0.0027 | Non-normal |
| ≥24 kg/m² | Abdomen | 90 | 0.8617 | 0.1250 | Normal |
| ≥24 kg/m² | Abdomen | 120 | 0.8908 | 0.2381 | Normal |
| ≥24 kg/m² | Love Handles | 30 | 0.8823 | 0.1981 | Normal |
| ≥24 kg/m² | Love Handles | 60 | 0.4703 | <0.0001 | Non-normal |
| ≥24 kg/m² | Love Handles | 90 | 0.5021 | <0.0001 | Non-normal |
| ≥24 kg/m² | Love Handles | 120 | 0.9377 | 0.5888 | Normal |
| ≥24 kg/m² | Thigh | 30 | 0.7979 | 0.0272 | Non-normal |
| ≥24 kg/m² | Thigh | 60 | 0.8656 | 0.1365 | Normal |
| ≥24 kg/m² | Thigh | 90 | 0.9153 | 0.3928 | Normal |
| ≥24 kg/m² | Thigh | 120 | 0.9422 | 0.6331 | Normal |
| ≥24 kg/m² | Forearm | 30 | 0.9855 | 0.9848 | Normal |
| ≥24 kg/m² | Forearm | 60 | 0.6698 | 0.0010 | Non-normal |
| ≥24 kg/m² | Forearm | 90 | 0.9485 | 0.6958 | Normal |
| ≥24 kg/m² | Forearm | 120 | 0.9440 | 0.6505 | Normal |
| <24 kg/m² | Abdomen | 30 | 0.5777 | 0.0001 | Non-normal |
| <24 kg/m² | Abdomen | 60 | 0.4967 | <0.0001 | Non-normal |
| <24 kg/m² | Abdomen | 90 | 0.5488 | <0.0001 | Non-normal |
| <24 kg/m² | Abdomen | 120 | 0.6772 | 0.0012 | Non-normal |
| <24 kg/m² | Love Handles | 30 | 0.9514 | 0.7250 | Normal |
| <24 kg/m² | Love Handles | 60 | 0.5070 | <0.0001 | Non-normal |
| <24 kg/m² | Love Handles | 90 | 0.9131 | 0.3763 | Normal |
| <24 kg/m² | Love Handles | 120 | 0.8415 | 0.0780 | Normal |
| <24 kg/m² | Thigh | 30 | 0.7745 | 0.0152 | Non-normal |
| <24 kg/m² | Thigh | 60 | 0.5140 | <0.0001 | Non-normal |
| <24 kg/m² | Thigh | 90 | 0.9601 | 0.8113 | Normal |
| <24 kg/m² | Thigh | 120 | 0.8740 | 0.1650 | Normal |
| <24 kg/m² | Forearm | 30 | 0.9153 | 0.3929 | Normal |
| <24 kg/m² | Forearm | 60 | 0.6007 | 0.0002 | Non-normal |
| <24 kg/m² | Forearm | 90 | 0.8688 | 0.1467 | Normal |
| <24 kg/m² | Forearm | 120 | 0.4667 | <0.0001 | Non-normal |
| W: Shapiro–Wilk statistic | | | | | |

**Table S3**. Per-subject and mean time of peak THb content in the SAT of different anatomic regions.

| Subject | Group | Abdomen | Love handles | Thigh | Forearm |
| --- | --- | --- | --- | --- | --- |
|  |  | **Peak time (min)** | **Peak time (min)** | **Peak time (min)** | **Peak time (min)** |
| 1 | Low BMI | 120 | 60 | 60 | 30 |
| 2 |  | 60 | 120 | 60 | 60 |
| 3 |  | 60 | 30 | 60 | 90 |
| 4 |  | 60 | 60 | 60 | 60 |
| 5 |  | 30 | 60 | 60 | 30 |
| 6 |  | 60 | 60 | 60 | 120 |
| 7 |  | 30 | 60 | 30 | 60 |
| 8 |  | 60 | 60 | 60 | 60 |
| Mean peak time | | **60 min** | **63.8 min** | **56.3 min** | **63.8 min** |
| 9 | High BMI | 60 | 60 | 90 | 60 |
| 10 |  | 90 | 60 | 30 | 60 |
| 11 |  | 60 | 60 | 60 | 60 |
| 12 |  | 60 | 30 | 60 | 120 |
| 13 |  | 60 | 60 | 90 | 60 |
| 14 |  | 90 | 30 | 60 | 0 |
| 15 |  | 60 | 60 | 60 | 30 |
| 16 |  | 60 | 60 | 60 | 60 |
| Mean peak time | | **67.5 min** | **52.5 min** | **63.8 min** | **56.3 min** |

**Table S4**. Changes over time of THb-content in the SAT of different anatomic regions.

| Position | Time (min) | Low BMI | High BMI | p-value  (*<0.05, **<0.01) |
| --- | --- | --- | --- | --- |
|  |  | **Median ± MAD (%)** | **Median ± MAD (%)** |  |
| Abdomen | 30 | 19.44 ± 10.07 | 12.87 ± 4.40 | 0.1415 |
|  | 60 | 64.20 ± 36.29 | 30.15 ± 10.20 | *0.0357 |
|  | 90 | 1.31 ± 20.02 | 9.96 ± 5.51 | 0.9164 |
|  | 120 | 3.48 ± 29.30 | -3.99 ± 17.83 | 0.2076 |
| Love handles | 30 | 52.85 ± 10.97 | 19.63 ± 9.44 | *0.0274 |
|  | 60 | 81.24 ± 22.93 | 38.12 ± 8.83 | *0.0157 |
|  | 90 | 19.13 ± 14.17 | 15.85 ± 13.43 | 1.0000 |
|  | 120 | 16.83 ± 6.29 | 7.29 ± 12.19 | 0.2076 |
| Thigh | 30 | 30.10 ± 9.86 | 14.43 ± 5.45 | *0.0274 |
|  | 60 | 53.86 ± 14.59 | 21.44 ± 3.69 | **0.0008 |
|  | 90 | 15.86 ± 8.62 | 12.49 ± 6.44 | 0.3171 |
|  | 120 | 18.95 ± 10.41 | 3.93 ± 4.22 | 0.5623 |
| Forearm | 30 | 54.96 ± 56.10 | -5.06 ± 28.17 | *0.0415 |
|  | 60 | 98.81 ± 66.10 | 24.10 ± 4.43 | *0.0357 |
|  | 90 | 14.35 ± 51.86 | 0.64 ± 27.61 | 0.1016 |
|  | 120 | 49.83 ± 35.88 | -13.49 ± 13.18 | *0.0157 |
| Abdomen-Thigh  (RDCM) | 30 | -1.07 | -0.32 |  |
|  | 60 | +0.41 | +0.82 |  |
|  | 90 | -1.01 | -0.42 |  |
|  | 120 | -0.78 | -0.72 |  |
| RDCM (Relative difference considering MADs): $\boldsymbol{(}\boldsymbol{media}\boldsymbol{n}_{\boldsymbol{1}}\boldsymbol{-media}\boldsymbol{n}_{\boldsymbol{2}}\boldsymbol{)/(}\frac{\boldsymbol{MA}\boldsymbol{D}_{\boldsymbol{1}}\boldsymbol{+MA}\boldsymbol{D}_{\boldsymbol{2}}}{\boldsymbol{2}}\boldsymbol{)}$ | | | | |

**Table S5.** Longitudinal analyses of capillary blood during the oral glucose test.

|  | Time (min) | Low BMI | High BMI | p-value  *<0.05 |
| --- | --- | --- | --- | --- |
|  |  | **Median ± MAD (%)** | **Median ± MAD (%)** |  |
| Glucose | 30 | 55.27 ± 19.84 | 67.18 ± 29.91 | 0.4622 |
|  | 60 | 33.78 ± 23.28 | 60.35 ± 35.54 | 0.2076 |
|  | 90 | 16.66 ± 16.08 | 15.32 ± 16.71 | 1.0000 |
|  | 120 | -2.19 ± 9.70 | -11.30 ± 8.33 | 0.8336 |
| Lactate | 30 | 91.89 ± 25.00 | 65.00 ± 25.33 | 0.3446 |
|  | 60 | 87.30 ± 38.71 | -10.42 ± 33.55 | *0.0209 |
|  | 90 | 59.97 ± 40.82 | 28.12 ± 54.78 | 0.5257 |
|  | 120 | 29.01 ± 31.01 | -10.00 ± 20.24 | 0.1996 |
| Cholesterol | 30 | -1.09 ± 5.02 | -3.37 ± 2.52 | 0.6277 |
|  | 60 | -3.33 ± 3.88 | -0.26 ± 2.05 | 0.8538 |
|  | 90 | -0.49 ± 6.34 | -3.60 ± 4.41 | 0.1853 |
|  | 120 | -0.99 ± 4.60 | -4.15 ± 4.12 | 0.2185 |
| Triglycerides | 30 | 0.00 ± 21.18 | 8.97 ± 18.12 | 0.1936 |
|  | 60 | -17.65 ± 20.50 | 5.24 ± 11.22 | 0.1476 |
|  | 90 | -17.65 ± 17.65 | -1.67 ± 19.34 | 0.2377 |
|  | 120 | -17.65 ± 10.92 | 0.00 ± 24.38 | 0.1511 |
